# A flexible behavioral method for measuring human and artificial intelligence alignment using representational similarity analysis

**DOI:** 10.1016/j.isci.2026.116400

**Published:** 2026-06-23

**Authors:** Mattson Ogg, Ritwik Bose, James Scharf, Christopher R. Ratto, Michael Wolmetz

**Affiliations:** 1 Research and Exploratory Development Department, Johns Hopkins University Applied Physics Laboratory, Laurel, MD 20723, USA

**Keywords:** applied sciences, computing methodology, artificial intelligence, natural language processing, social sciences, linguistics

## Abstract

As we consider entrusting large language models (LLMs) with key societal and decision-making roles, measuring their alignment with human cognition becomes critical. This requires methods that can assess how these systems represent information and facilitate comparisons with human understanding across diverse tasks. To meet this need, we adapted representational similarity analysis (RSA), using pairwise ratings to help quantify alignment between AIs and humans. Among the models we studied, GPT-5-mini and Claude Sonnet 4.5 showed the strongest alignment with human text ratings. Llama-4 was the best aligned open-source model. However, gaps between LLM and human behavior remain. No model we studied adequately captured the inter-individual variability observed among human participants, and models only moderately aligned with individual human responses. We demonstrate the utility of this approach across multiple modalities (words, sentences, and images), helping further our understanding of how LLMs encode knowledge, and enabling an examination of alignment with human cognition.

## Highlights

• Human-AI alignment was measured via pairwise ratings and RSA across text and images
• High group-level alignment was observed for GPT-5-mini, Claude Sonnet, and Llama-4
• Lower alignment was found for image ratings, but better cross-modal alignment
• LLMs responded very consistently and no model captured human individual variability

## Graphical abstract

**Figure:**
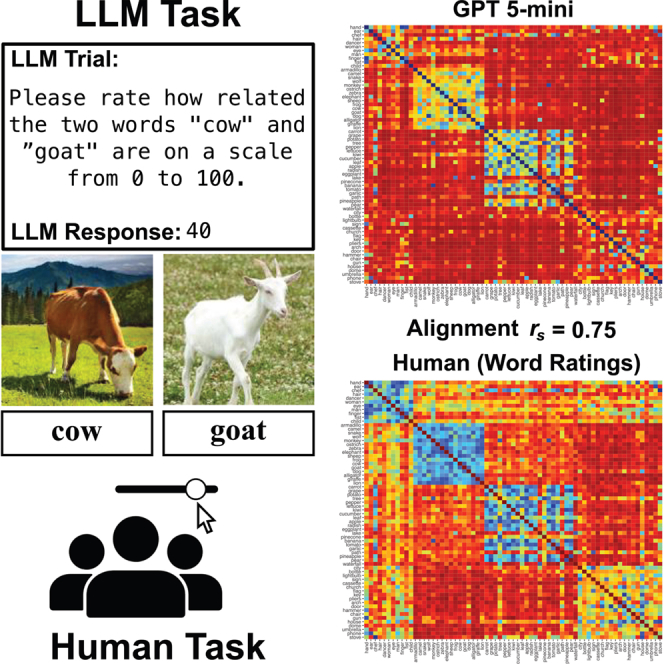

## Introduction

Foundation model reasoning^1^^,^^2^ and perceptual skills^3^^,^^4^ may soon match or exceed human performance across a wide range of tasks.^5^^,^^6^^,^^7^ The rapid pace of this progress, as exemplified by large language models (LLMs),^8^^,^^9^ has initiated a discussion of whether and how these models should be integrated into everyday life or given additional responsibilities.^10^ The increasing deployment of artificial intelligence (AI) systems in critical roles (and the potential for replacing humans in those roles) requires scalable, generalizable methods for measuring how FMs represent knowledge about the world, and for evaluating how those representations and downstream behaviors compare to complex human behaviors (see recent discussion^11^). The transition from fundamentally narrow models of moderate size and complexity to increasingly general models that are larger and more complex compounds the classic opacity problem of deep neural networks: discerning how an LLM processes a given input or arrives at a decision has never been as challenging or as critical as it is now. Fortunately, cognitive science and psychological research has focused on this exact challenge in the context of biological intelligence and serves as a productive framework for studying artificial cognition and its alignment with human cognition.

As an exemplar of this emerging framework, *Turing experiments* capitalize on the rich history of experimental psychology to measure the cognitive and behavioral alignment between human and artificial intelligence.^12^^,^^13^ In a Turing experiment, an LLM is used to simulate a sample of the human population over repeated runs,^13^^,^^14^ sometimes using simulated participant identities for each new run,^12^^,^^15^ and is prompted to engage in classic psychology tasks (e.g., the prisoner’s dilemma, the ultimatum game, or the Milgram shock experiment). While still very early, this line of work has provided insight into LLM reasoning and has highlighted similarities with human behavior. For example, the GPT family of models (as well as different open-source models) can exhibit discrete personalities based on their prompting^14^ that influence behavior.^16^ These models can also generate responses in behavioral tasks that fall within the range of human variability,^13^ and, finally, larger, more recently developed language models better align with human behavior.^12^ This approach allows for an examination both of how similar LLM behavior is to human behavior in the aggregate (i.e., using the central tendency of a sample of simulated participants) but can also help us understand inter-individual variability among human and AI participants. These initial studies suggest there could be value in using other paradigms and techniques to explore the knowledge and behavior of LLMs and how they align with human knowledge and behavior. There are active discussions regarding how best to use this framework to support psychological research and how, if executed properly, LLMs can help further our understanding of human cognition.^17^^,^^18^ We note, however, that here we are interested in using behavioral testing paradigms borrowed from cognitive science to improve our understanding of the cognition of LLMs,^19^ rather than humans.

One of the most productive methods for mapping the structure of how an individual represents information about the world is the use of pairwise ratings of similarity (or dissimilarity) with respect to a pair of stimuli.^20^^,^^21^^,^^22^ This class of tasks is adaptable to a wide array of domains and questions (e.g., “How similar are the words ‘apple’ and ‘hand?’” or “How similar are these two images?”), and is most useful when the experimenter does not have direct access to a participant’s internal representations (i.e., neuronal activations or embeddings), as is the case in traditional psychophysics and cognitive science experiments as well as for many frontier LLMs. Ratings elicited by participants on each trial comprise a behavioral distance metric for the two stimuli that were presented. These ratings can be organized into a symmetrical matrix whose rows and columns correspond to the probe items used in each rating trial and can be analyzed using techniques like multi-dimensional scaling to visualize the geography of how different items relate to one another^23^ or to test different hypotheses.^24^ This approach has deeply informed a range of questions in human perception and cognition including object relations^25^^,^^26^ and semantic information,^27^ as well as musical pitch^28^ and timbre.^29^^,^^30^

Representational similarity analysis (RSA^31^) builds on the use of distance, or dissimilarity, matrices (“DSMs” including from pairwise ratings) to quantify the similarity of *representational spaces* among diverse systems: across organisms,^32^ individuals,^33^ models,^34^^,^^35^ or biological substrates such as different brain regions.^27^^,^^36^^,^^37^ In RSA, the organized distance matrices are correlated with one another to quantify the agreement of the pairwise ratings (or distances) between each system.^38^ That is, RSA can be used to quantify how similarly two species (e.g., humans and primates^32^) process object images at different stages of the visual hierarchy, to align object representations from different neuroimaging modalities across time and cortical space,^39^ or to investigate how the computations performed by layers of convolutional networks trained for visual object recognition relate to the computations of the ventral visual pathway.^40^

RSA has also been used to understand the representations of neural network models (e.g., by correlating distances derived from model embeddings^11^^,^^34^^,^^35^^,^^41^^,^^42^). However, for current LLMs, these embeddings are not always accessible. Instead, the representational structure of these models can be distilled by formulating this analysis as a Turing experiment where the model is queried with pairs of stimuli and asked to provide a similarity rating for each pair. A helpful advantage of combining pairwise ratings with RSA is that it enables comparisons between any systems capable of producing comparable behavioral outputs, without requiring access to or assumptions about their internal representations. This makes it well suited to comparing human and artificial intelligence, where internal processing mechanisms may be fundamentally different or inaccessible.

A growing body of work has begun to adapt pairwise rating methods to behaviorally probe LLMs and to measure their alignment with humans. A series of studies by Marjieh and colleagues^3^^,^^43^^,^^44^ have explored a continuous approach to mapping LLM knowledge via representational distances either from model embeddings or model ratings. Their initial results found that LLMs can predict human similarity judgments across multiple perceptual domains based on text input alone. Dickson and team^45^ asked similar questions regarding perception based on visual input, finding that different LLM models aligned with human ratings along some (but not all) perceptual dimensions. Finally, Du and colleagues^46^ studied LLM and VLM perception via a slightly different leave-one-out oddball task similar to previous work with human raters,^47^ finding high alignment with human ratings and neural responses. However, these analyses were primarily focused on visual object ratings and RSA analyses were focused primarily on model embeddings or neural responses rather than directly measuring pairwise rating behavior. None of these previous studies have compared representations across text and image domains or undertook an evaluation of the variability among ratings of LLM participants.

We build on this prior work at the intersection of Turing experimentation and RSA by designing a behavioral pairwise rating task to probe the knowledge and behavior of LLM agents as a strategy for measuring alignment between artificial and biological intelligence across information domains (e.g., text and images). This approach does not require access to internal model representations and can provide a behavioral readout for different modalities that helps enable direct comparison with human data. Using this method, we measure the relationship between a collection of LLM and human judgments for specific sets of well-studied probe objects via words and images. In the process, we demonstrate the flexibility of this approach to facilitate comparisons within and across different modalities (i.e., text and images), quantify individual variability among LLMs and humans, and evaluate prompt effects among standard Turing experiment formulations. This approach complements accuracy-based benchmark tasks and other model interpretation methods, helping to shed light on LLM cognition through the lens of LLM alignment with human behavior.

## Results

### Word similarity judgments

**Figure 1:**
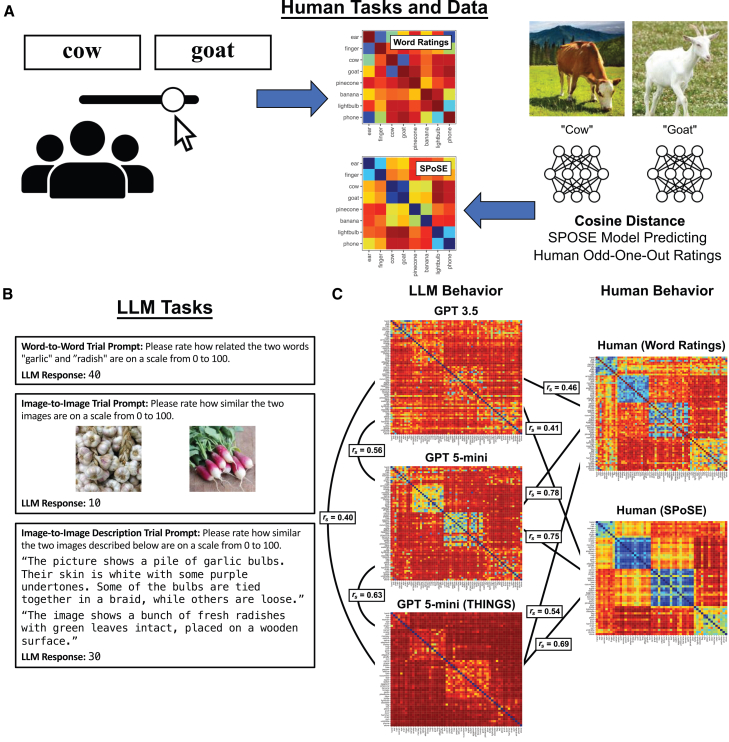
Summary of behavioral pairwise ratings, RSA methodology and results (A) Depiction of the task and models used to derive pairwise human data via word ratings (left) and a model of human responses on an odd-one-out task for images, called SPoSE (right). (B) Synopsis of a pairwise rating behavioral RSA trial for each of our main experiments. LLM responses for each trial are entered into the intersecting cell for the item pair in the corresponding DSMs. (C) DSMs for LLMs (*n* = 24 in each matrix) and humans averaged over participants (*n* = 8 for word ratings). The Spearman rank correlation values between them summarize their representational alignment (all Bonferroni-corrected *p* < 0.05). See text and Tables S2 and S3 for additional details as well as Supplemental Methods S1, S2, and S3 in the supplemental information for a depiction of the full prompts for each experiment.

We designed a prompt-based task to map the high-level semantic representational space of LLMs, drawing inspiration from the power and ostensible simplicity of pairwise rating tasks (Figure 1A, similar to other work^3^). Even without direct access to the model’s embeddings or internal representations, the flexibility of the chat prompt interface allowed us to repeatedly query the model with questions asking the LLM to rate the similarity of two concepts (see Figure 1B and Supplemental Methods S1 in the supplemental information; for information on costs and runtime see Supplemental Methods S4 in the supplemental information). This method also allowed us to quantify the representational similarity (or representational alignment) of two model systems, and to specifically assess how similar LLM semantic representations are to those of humans. Importantly, our goal for this initial effort was not specifically to rank models, but rather to demonstrate a generalizable framework for measuring alignment.

Repeated runs for a given model allowed us to examine the variability of LLM responses by assigning the model a different simulated *participant* identity for it to assume on each new run.^12^^,^^13^^,^^14^^,^^15^ The LLM was assigned these identities (for example, “Ms. Olson”) each time it was initialized for a given participant run: both when introducing the task and during the subsequent run of rating trials (see Supplemental Methods S1 in the supplemental information).

To validate this approach, we compared LLM responses to previously collected pairwise ratings of semantic relatedness for different object concepts provided by a cohort of human raters. In the original study, eight participants judged the semantic relatedness of all pairs of 67 object concepts, originally collected for comparison with neural responses to assess when semantic meaning emerges within the human ventral visual pathway (described in the original publication^27^ see Figure 1C; Table S1). We note that while this sample of human data was valuable for assessing links between human behavior and neural representations,^27^ and for developing our method, future work should be conducted on a larger sample of participants and stimuli. The same task instructions and word stimuli were presented to different commercial and open-source LLMs (including smaller open-source language models that were run locally) to elicit similarity ratings that could be compared to human ratings (Figure 1B).

Ratings from commercial models GPT-5-mini (*r*_*s*_ = 0.776) and Claude Sonnet 4.5 (*r*_*s*_ = 0.765) were most similar to the human participant responses among the models that were tested (all Bonferroni-corrected *p* < 0.05 unless otherwise stated, see Figure 1C; Table S2 for more details and for the full set of comparisons, see Figure S1 for a full gallery of group-level model DSMs illustrating these patterns). Among smaller, open-source language models and language model embeddings, Gemma-2-9b ratings (*r*_*s*_ = 0.658), Solar-10.7b (*r*_*s*_ = 0.648), Llama-3-8b (*r*_*s*_ = 0.586), Phi-4-14b (*r*_*s*_ = 0.638), Mistral-7b (*r*_*s*_ = 0.532), GloVe embeddings (*r*_*s*_ = 0.643) and Ada embeddings (*r*_*s*_ = 0.437) were closely aligned with human ratings. In many cases these smaller LLMs were more aligned with human ratings than GPT-3.5 (*r*_*s*_ = 0.456). Ratings from Llama-4 (*r*_*s*_ = 0.712), another larger open-source model, were also very highly aligned with human ratings, outperforming GPT-4 (*r*_*s*_ = 0.696) but not GPT-4o (*r*_*s*_ = 0.740) or GPT-4o-mini (*r*_*s*_ = 0.736). In almost all cases, larger or newer versions of models out performed older ones. However, two exceptions to this were Gemma-3-12b (*r*_*s*_ = 0.619) and the full-size GPT-5.2 (*r*_*s*_ = 0.706), which under-performed previous generation models (Gemma-2, GPT-4o) as well as smaller configurations from the same generation (GPT-5-mini, GPT-5-nano; *r*_*s*_ = 0.743), respectively. Llama-2 (Llama-2-7b, *r*_*s*_ = 0.245; Llama-2-uncensored-7b, *r*_*s*_ = 0.201) and BERT (bert-base-uncased, *r*_*s*_ = 0.188) models had the lowest alignment with human ratings among the text models that were tested (the albert-xxlarge-v2 alignment of *r*_*s*_ = -0.001 did not survive Bonferroni correction).

The stimuli in these experiments were organized into discrete object categories (human, animal, natural, and man-made objects, see Table S1), which allowed us to examine how these models represented different aspects of within- and between-category semantic structure. Most models were more aligned with human participant ratings of items within the same object category (GPT-3.5, *r*_*s*_ = 0.472; GPT-4, *r*_*s*_ = 0.780; GPT-4o-mini, *r*_*s*_ = 0.747; GPT-4o, *r*_*s*_ = 0.781; GPT-5-nano, *r*_*s*_ = 0.840; GPT-5-mini, *r*_*s*_ = 0.859; GPT-5.2, *r*_*s*_ = 0.814; Claude Haiku 4.5, *r*_*s*_ = 0.784; Claude Sonnet 4.5, *r*_*s*_ = 0.856; Gemma-7b, *r*_*s*_ = 0.479; Gemma-2-9b, *r*_*s*_ = 0.691; Gemma-3-12b, 0.659; Gemini-2.5-Flash-Lite, 0.702; Llama-3-8b, *r*_*s*_ = 0.613; Llama-3.1-8b, *r*_*s*_ = 0.484; Llama-4, *r*_*s*_ = 0.787; Phi-3-medium-14b, *r*_*s*_ = 0.498; Phi-4-14b, *r*_*s*_ = 0.614; Mistral-7b, *r*_*s*_ = 0.482; Solar-10.7b, *r*_*s*_ = 0.715; GloVe, *r*_*s*_ = 0.801; Ada, *r*_*s*_ = 0.660, all Bonferroni-corrected *p* < 0.05), compared to items from different categories (GPT-3.5, *r*_*s*_ = 0.293; GPT-4, *r*_*s*_ = 0.511; GPT-4o-mini, *r*_*s*_ = 0.601; GPT-4o, *r*_*s*_ = 0.598; GPT-5-nano, *r*_*s*_ = 0.552; GPT-5-mini, *r*_*s*_ = 0.626; GPT-5.2, *r*_*s*_ = 0.498; Claude Haiku 4.5, *r*_*s*_ = 0.437; Claude Sonnet 4.5, *r*_*s*_ = 0.608; Gemma-7b, *r*_*s*_ = 0.389; Gemma-2-9b, *r*_*s*_ = 0.474; Gemma-3-12b, 0.422; Gemini-2.5-Flash-Lite, 0.458; Llama-3-8b, *r*_*s*_ = 0.377; Llama-3.1-8b, *r*_*s*_ = 0.411; Llama-4, *r*_*s*_ = 0.548; Phi-3-medium-14b, *r*_*s*_ = 0.481; Phi-4-14b, *r*_*s*_ = 0.489; Mistral-7b, *r*_*s*_ = 0.466; Solar-10.7b, *r*_*s*_ = 0.471; GloVe, *r*_*s*_ = 0.485; Ada, *r*_*s*_ = 0.246, all Bonferroni-corrected *p* < 0.05; V = 336, *p* < 0.001, two-sided paired Wilcoxon signed rank exact test for within- vs. between-category text model alignment). In general, the models that were better aligned with human participant ratings were most aligned with respect to within-category structure and their between-category structure was sparse by comparison (i.e., high performing models were more aligned when rating objects from the same category such as “cow” and “goat” than when rating objects from different categories like “cow” and “phone” and these between-category ratings tended to be near zero; see Table S2; Figure S1). This indicates that there is some nuance in how human raters represent some between-category semantic relations that is not captured well by the LLMs.

### Image similarity judgments

Initial experiments demonstrated that our pairwise rating task allowed us to behaviorally probe the structure of an LLM’s semantic representations and compare those with humans. Next, we evaluated the generalizability of this method for comparing human and LLM behavioral judgments across domains. For this we used the images corresponding to the object words rated in the study by Carlson and colleagues^27^ (obtained from Cichy, Pantazis, and colleagues,^48^ although they originated from Kriegeskorte and colleagues^32^) referred to as the “Carlson-Image” stimuli (the human behavior ratings were collected to analyze neural responses to these images). We ran an additional set of experiments using corresponding images from the THINGS database.^49^^,^^50^ This complemented the Carlson-image stimulus set, which comprises a cropped view of each object presented on a gray background, with more natural depictions of each object (including backgrounds) typical of the THINGS dataset (see discussion^51^). There was incomplete overlap in the object classes between these two stimulus sets, so analyses were restricted to models or responses for the 55 object classes present in both the Carlson-Image stimulus set and the THINGS database (see Table S1).

We adapted the text-based pairwise rating task to elicit similarity ratings for pairs of images from models capable of handling both text and image inputs (GPT-4-Vision, GPT-4o, GPT-4o-mini, GPT-5.2, GPT-5-mini, Gemini-2.5-Flash-Lite, Claude Sonnet 4.5, and Llama-4; see Figure 1B and Supplemental Methods S2). For a comparison with human behavioral ratings, we used cosine distances between sparse positive similarity embeddings (SPoSEs) generated for each object.^47^ These embeddings were learned so as to accurately predict odd-one-out behavioral judgments for a large number of over 1,800 objects represented by a large set of images from the THINGS database. LLM ratings were also compared with representations derived from a popular high performing deep convolutional network (AlexNet) trained on either the ImageNet database (referred to as AlexNet-LSVRC2012) or a more ecologically representative dataset (referred to as AlexNet-Ecoset; both from the same original publication,^41^ see Figure S1).

We found that GPT-5-mini predicted SPoSE model distances (i.e., a model of human visual image ratings) reasonably well (*r*_*s*_ = 0.619 based on the Carlson-image stimuli; *r*_*s*_ = 0.690 based on the THINGS stimuli; all Bonferroni-corrected *p* < 0.05; Table S3), but overall the image-processing models aligned slightly less with human behavior than their text-only counter parts (W = 127, *p* < 0.001, two-sided Wilcoxon Rank-Sum test between text model alignment with human data and vision model alignment and SPoSE model distances; see Tables S2 and S3). The open-source Llama-4 model also aligned well with SPoSE model distances (*r*_*s*_ = 0.564 based on the Carlson-image stimuli; *r*_*s*_ = 0.567 based on the THINGS stimuli; all Bonferroni-corrected *p* < 0.05). For most models (except GPT-5.2), alignment was slightly better for the THINGS stimuli (V = 33, *p* < 0.05, two-sided paired Wilcoxon signed rank exact test), perhaps due to the more natural depictions of each image in that dataset (e.g., including natural backgrounds), which could have been a better match to the training data. The AlexNet-Ecoset models were less well aligned than most visual processing LLM model ratings, but otherwise aligned reasonably well with the model of human behavior based on THINGS stimuli (*r*_*s*_ = 0.564), better than the Carlson-image stimuli (*r*_*s*_ = 0.407), and overall better predicted SPoSE distances than AlexNet-LSVRC2012 (based on THINGS, *r*_*s*_ = 0.428; based on Carlson-image, *r*_*s*_ = 0.372).

RSA facilitates comparisons across input domains (like vision and language), which are useful for assessing the potential for modality-agnostic conceptual representations (which is considered to be a central feature of semantic knowledge^52^^,^^53^). For example, human ratings of these objects via *text* were well aligned with the SPoSE model distances, which were based on images (*r*_*s*_ = 0.729).

Visual processing LLMs were less well aligned across text and image domains (for example, GPT-5-mini text-ratings correlated with Carlson-image ratings, *r*_*s*_ = 0.478 and with THINGS images, *r*_*s*_ = 0.625; see Table S3). Notably, GPT-5-mini ratings of these object *words* aligned better (*r*_*s*_ = 0.749) with SPoSE ratings (which were derived based on images) than the GPT-5-mini model’s ratings of images, as did other text models including GPT-5-nano (*r*_*s*_ = 0.706), Claude Sonnet 4.5 (*r*_*s*_ = 0.697; both based on word ratings). In some cases, model alignment with human pairwise *text* ratings increased given the reduced 55-item stimulus set that accommodated the THINGS dataset classes, but where possible we defer to the results of the larger 67-item sample.

Similar to the text experiments, Human-LLM alignment for images was explored with respect to within- and between-category ratings. Again, models were decidedly more aligned with human ratings for within-category comparisons than for between-category comparisons (V = 136, *p* < 0.001, two-sided paired Wilcoxon signed rank exact test for within- vs. between-category GPT vision model alignment). AlexNet models were less aligned to SPoSE (human behavior) for both within-category (AlexNet-Ecoset for Carlson-image, *r*_*s*_ = 0.109; AlexNet-Ecoset for THINGS, *r*_*s*_ = 0.469; AlexNet-LSVRC2012 for Carlson-image, *r*_*s*_ = 0.080; AlexNet-LSVRC2012 for THINGS, *r*_*s*_ = 0.316, with only the THINGS analyses surviving multiple comparison correction) and between-category structure (AlexNet-Ecoset for Carlson-image, *r*_*s*_ = 0.051; AlexNet-Ecoset for THINGS, *r*_*s*_ = 0.231; AlexNet-LSVRC2012 for Carlson-image, *r*_*s*_ = 0.060; AlexNet-LSVRC2012 for THINGS, *r*_*s*_ = 0.024, with only the comparison with the AlexNet-Ecoset representations on the THINGS dataset surviving multiple comparison correction).

### Increasing human-LLM alignment through prompting and hyperparameters

**Figure 2:**
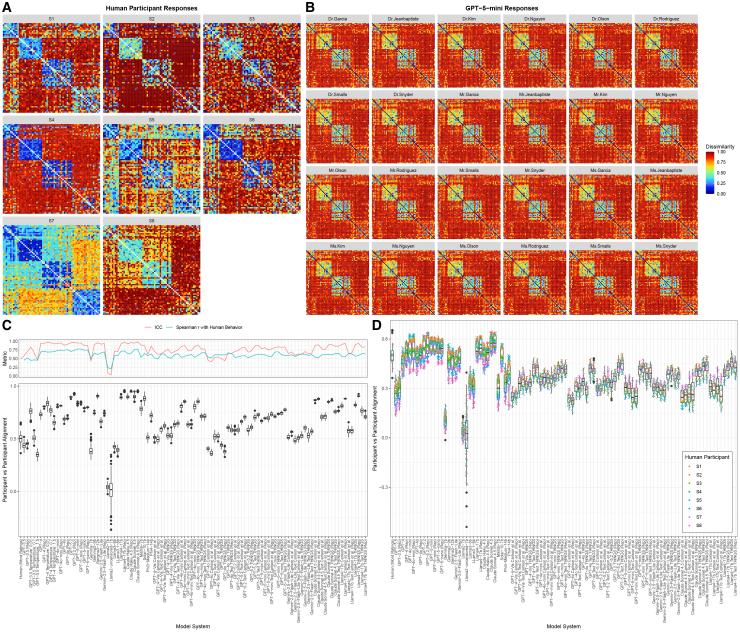
Quantifying variability among participants for each model system (A) DSMs for each of the human participants who provided word-similarity ratings. (B) DSMs for a cohort of GPT-5-mini participants. (C) Summary of variability among individuals of each model system, (top) plotting aggregate ICC values and group-level Spearman correlation statistics across each set of real and simulated participants (*r*_*s*_ = 0.544, *p* < 0.001 between ICC and alignment statistics), and (bottom) a plot of the distribution of inter-subject representational alignment for each model system. Temperature was set to 1.0 unless otherwise stated. (D) individual-level cross-system alignment between individual LLM and human participants. Alignment is organized by model system along the *x* axis and colored points indicate the human participant in the comparison. The models and experiments are grouped to facilitate easy comparison: temperature experiments are grouped together on the left side of (C) and (D), while different prompting experiments are grouped beside one another throughout (C) and (D). The *x* axis labels convey the information for each experiment.

LLMs provide increasingly accurate proxies for human ratings at the group level, especially for text-based tasks. However, there is interest in methods to increase the similarity between the representational and behavioral spaces of LLMs and humans,^11^ and there is room for improvement for even the most well-aligned LLMs observed in our study (see Tables S2 and S3). Thus, we undertook an additional set of experiments that explored ways to increase alignment between LLM and human behavior via changes to hyperparameters and model prompts. GPT models were the focus for these experiments because of their popularity and overall good alignment performance. The results of all of these experiments and their relationship to human data, and other hyperparameters can be seen in Figures 2C and 2D. Temperature experiments are grouped on the left-hand side, while different prompting experiments are grouped beside one another.

First, we investigated increasing the alignment of image processing LLMs. These models achieved modest alignment with human behavior, and the text-only ratings of these object concepts were often more similar to models of human visual semantic behavior. Therefore, relying more heavily on their text processing capabilities may increase alignment with human behavior. To test this, a new set of LLM participants was run for the image rating task, where each LLM participant first provided a description of each image. Then, the LLM participants were asked to make their pairwise ratings based on their own *descriptions* of the images they had just provided (see Figure 1B and Supplemental Methods S3). In other words, the images were first converted to text descriptions, and similarity judgments were made based on these text descriptions. The results of these experiments are also indicated in Tables S2 and S3 (denoted, e.g., “GPT-5-mini-Vision Descriptions” or “Vis. Desc.”). Rating text descriptions in this way increased alignment between each visual processing LLM and the SPoSE model of human visual semantics for both stimulus sets (V = 110, *p* < 0.05, two-sided paired Wilcoxon signed rank exact test comparing LLM image ratings and SPoSE model alignment with LLM text description ratings and SPoSE model alignment).

Next, we assessed whether different approaches to operationalizing LLM participants impacted alignment results. Aher and colleagues^12^ ascribed a surname and honorific to each new LLM participant, and other experiments have simply re-queried the model without explicitly assigning an identity, and still obtained a distribution of human-like responses^3^^,^^13^^,^^14^ or did not find substantial differences among individual-level prompts for improving alignment with human behavior.^15^ To examine what effect this has on LLM responses and the semantic representation distances elicited by our RSA experiment, we re-ran our experiments for the GPT models with all surnames and honorifics removed and measured group-level representational alignment. The results of these experiments are reported in Tables S2 and S3 (denoted by “[Repeats]” or “[Rep.]”). Removing surnames and honorifics increased alignment with human ratings for most of the text-only models tested (increase in GPT-3.5 alignment from *r*_*s*_ = 0.456 to *r*_*s*_ = 0.521; for GPT-4 from *r*_*s*_ = 0.696 to *r*_*s*_ = 0.708; for GPT-4o-mini from *r*_*s*_ = 0.736 to *r*_*s*_ = 0.746; for GPT-4o from *r*_*s*_ = 0.740 to *r*_*s*_ = 0.758; for GPT-5-nano from *r*_*s*_ = 0.743 to *r*_*s*_ = 0.748; for GPT-5-mini from *r*_*s*_ = 0.776 to *r*_*s*_ = 0.774; for GPT-5.2 from *r*_*s*_ = 0.706 to *r*_*s*_ = 0.738; for Claude Haiku 4.5 from *r*_*s*_ = 0.647 to *r*_*s*_ = 0.653; for Claude Sonnet 4.5 from *r*_*s*_ = 0.765 to *r*_*s*_ = 0.772; for Gemini-2.5-Flash-Lite from *r*_*s*_ = 0.662 to *r*_*s*_ = 0.696; for Llama-4 from *r*_*s*_ = 0.712 to *r*_*s*_ = 0.721; V = 1, *p* < 0.01, two-sided paired Wilcoxon signed rank exact test comparing LLM text model ratings and human ratings with and without surnames or honorifics). However, there were mixed results regarding whether this more minimal style of prompting improved alignment in the image rating experiments (see Table S3).

The temperature hyperparameter increases or decreases the verbosity and randomness of a LLM’s responses, and thus could influence LLM participant responses (as reported previously^14^) We explored the influence of this hyperparameter in our text-based experiments by re-running our GPT-3.5 and GPT-4 participants across a range of temperature settings: 0.01, 0.7, and 1.5 (combined with our results reported thus far which were run at 1.0). In general, this did not have a substantial or systematic influence on alignment (GPT-3.5 *r*_*s*_ = 0.451, 0.464, 0.456, 0.454; and GPT-4 *r*_*s*_ = 0.687, 0.692, 0.696, 0.699 across temperatures of 0.01, 0.7, 1.0 and 1.5, respectively). However, we noted changes in the consistency of responses across participants in these temperature-sweep experiments, which we address in the following section.

### Individual variability in human and LLM responses

Individual differences (or the variability observed between individuals) is a fundamental aspect of human behavior. Inter-individual variability was salient in our human behavioral data (for text-based ratings, Figure 2A), and overall led to only modest agreement among human raters. Thus, an accurate encapsulation of human behavior by a cohort of LLM participants would be able to achieve *both* a high alignment with human responses at the group level, and moderate inter-individual variability. Quantifying and matching these inter-individual differences with LLMs is critical for generating useful proxies of human behavior, but this has thus far been a less-explored dimension of human-AI alignment. For example, when viewing a cohort of GPT-5-mini participants’ responses (Figure 2B), that were highly aligned with human behavior when averaged together at the group-level, there is a stark contrast in terms of the homogeneity of LLM responses relative to human participants at the individual-level. To quantify this aspect of performance, alignment among the unique participants (human and simulated) within each experiment was calculated along with an intraclass correlation coefficient (ICC) among each set of participants. An additional analysis examined how well a given LLM participant might have aligned with an individual human participant (i.e., individual-level human-LLM alignment). We compare these measures alongside group-level representational alignment in Figure 2.

LLMs that achieved the best overall alignment with human data at the group level produced strikingly consistent response patterns and were much more consistent than the cohort of human raters (correlation between *r*_*s*_ for human behavior alignment and ICC in Figure 2C, *r*_*s*_ = 0.544, *p* < 0.001). The distribution of individual responses varied widely across models (Figure S2) Human data (both text ratings and SPoSE distances) had a slightly bimodal distribution of rating responses. This feature was somewhat captured by the GPT-4o, Gemma, and Gemini-2.5 responses but models generally lacked this characteristic. Changes in hyperparameters or prompting, particularly temperature, influenced inter-subject agreement via both ICC and inter-subject alignment (Figure 2C). However, there was only a small influence on group level alignment with human data. Overall the best performing commercial and open-source models (GPT-5-mini, Claude Sonnet 4.5, and Llama-4 were much more consistent in their responses than human participants. These models achieved strong group-level alignment with human judgments (GPT-5-mini, *r*_*s*_ = 0.774 to 0.776, *ICC* = 0.946 to 0.948; Claude Sonnet 4.5, *r*_*s*_ = 0.765 to 0.772, *ICC* = 0.945 to 0.980; Llama-4, *r*_*s*_ = 0.712 to 0.721, *ICC* = 0.928 to 0.970), which exceeded the range of individual human inter-subject correlations (*r*_*s*_ among pairs of individual human subjects ranged from 0.363 to 0.654, median 0.504, *ICC* = 0.490). GPT-3.5 (*ICC* = 0.582 to 0.708), GPT-4o ratings of images (*ICC* = 0.412 to 0.498) and Gemma-7b (*ICC* = 0.422) models were closer to capturing the inter-subject agreement of human participants (*ICC* = 0.490), but overall, their alignment with human behavior (at the group level) was lower (*r*_*s*_ = 0.502 to 0.606).

Finally, individual-level alignment between human and LLM participants was evaluated to understand how closely a given LLM participant might align with a given human participant’s behavioral ratings. Figure 2D displays the alignment between each LLM participant (organized by model system) and each human participant (colored points). The highest individual human-LLM alignment was observed between a Claude Sonnet 4.5 participant and human participant S5 (*r*_*s*_ = 0.649). However, overall, inter-individual cross-system alignment was lower than when responses from LLM and human participants were averaged (*r*_*s*_ = 0.649 best individual alignment compared to *r*_*s*_ = 0.765 for group-averaged Claude Sonnet 4.5 alignment). Similar patterns were observed for other model participants that aligned best with individuals (GPT-5.2 [Rep.], *r*_*s*_ = 0.643 best individual alignment with S1 compared to group-averaged *r*_*s*_ = 0.738; Llama-4 [Rep.], *r*_*s*_ = 0.623 best individual alignment with S2 compared to group-averaged *r*_*s*_ = 0.721). However, while no model seems to perfectly represent a *specific* human’s performance, the performance of a given model may be overall similar to *another* human: many LLM participants (GPT-4 and above, Gemma-2 and above, Gemini-2.5-Flast-Lite, Llama-4, Claude, Solar, etc.) aligned with individual human participants in a range that is similar to the alignment observed among individual human participants (*r*_*s*_ = 0.363 to 0.654). Participants from each model also appeared to occupy distinct spaces from one another when rating distances were visualized in a common low dimensional space (Figures S3 and S4). In general, no models overlapped with the human participant responses, which were mostly clustered tightly together (although GPT-5-mini and Llama-4 participants were nearby). Ultimately, data from a larger human sample should be used to draw stronger conclusions regarding individual-level human-LLM alignment. A larger sample of human participants might identify stronger individual-level human-LLM alignment and patterns of behavior across individuals for which LLMs might align best.

## Discussion

The performance of LLMs continues to rapidly improve, raising increasingly important questions about reliability, explainability and alignment with human objectives. If LLMs begin to be used widely as proxies for human behavior (potentially in simulations, as assistants or for human subjects testing), methods will be needed for assessing how human-like a given model’s behavior can be across a wide array of scenarios and for increasing alignment between LLMs and humans. We adapted a generalizable pairwise rating task, based around RSA as an additional tool to help probe the representational structure of LLMs that would otherwise be black-box interfaces. Experiments using this task for a wide-ranging but non-comprehensive set of models found that GPT-5-mini and Claude Sonnet 4.5 ratings conveyed a representational structure that is highly (but not perfectly) aligned with human semantic representational structure (obtained through the same behavioral pairwise rating task), especially when compared to other smaller models and when relying primarily on text processing capabilities (regardless of the input modality). Also, despite being smaller, many of the recent generation of compact 8- to 14-billion parameter open-source language models such as Llama-3, Phi-4, Gemma-2, and Solar were well aligned with human semantic ratings (more even than the substantially larger GPT-3.5). However, the inter-individual variability observed among humans was difficult to reproduce among LLM participants. Group-level alignment between LLMs and human behavior could be increased by changing some prompts and hyperparameters. Finally, human participants’ object ratings were more consistent across text and image modalities than cross-modal LLM ratings.

These studies extend prior work^3^^,^^43^^,^^44^^,^^45^ by examining multiple models, comparisons within and across stimulus domains, and by examining inter-individual variability all within the same framework using matched stimuli. Our use of a continuous subjective rating task rather than assessing accuracy or performance like many LLM evaluations^54^ provides a useful complement to standard practices. The pairwise rating method used here provides another tool to help probe nuanced, high-level features of knowledge representations and relationships among concepts in a flexible manner.

One unexpected finding was the high level of alignment between LLMs and humans evaluating *text* and the SPoSE representations of human ratings for *images*. This could be the result of overall weaker image processing capability of these models. However, another likely explanation could be that SPoSE embeddings are specific to overall object *categories*, representing human behavior over ratings of multiple images for a given object category, rather than representing nuances of specific images. At this more aggregated level, image and semantic representations may converge. The AlexNet image baseline models, which represent features of individual images (but were trained to classify object categories), did indeed align highly with GPT-5-mini and Claude Sonnet ratings of the same THINGS images (*r*_*s*_ = 0.568 to 0.609 for the Ecoset model), offering support that some LLM ratings took into account low-level visual features of the images. However, the AlexNet model also aligned almost as well with human *text* ratings (*r*_*s*_ = 0.572), which were not based on any explicit visual information. The AlexNet Ecoset model was also more aligned with human text ratings than most other LLM ratings of images, including SPoSE distances (*r*_*s*_ = 0.564). This study was not set up explicitly to adjudicate these issues, but our initial results invite interesting future experiments (using similar pairwise rating approaches and other methods) that could help better understand cross-modal object representations for these intelligent systems.

Human and LLM participants exhibited stark differences in their ratings of items from different categories. Most high performing models captured functional relationships among items that spanned object categories (chef/stove, chef/garlic, ear/phone, monkey/banana, etc.), but overall, they rated compositional relationships as less related (eye, ear, and finger were mostly rated as similar to most animals by humans, but not by the LLMs). Some of this can be seen in a band of more similar ratings overall between the human and body part categories (upper left of the DSM) and others (bright bands extending from the upper left across categories) that were largely absent among the LLMs (Figure S1), though Llama-4 and GPT-5-mini captured this feature slightly better. This could indicate some hierarchical or relational features humans took into account that LLMs mostly ignored, and might account for the overall low between-category alignments we observed. We used a well understood but ultimately small sample of stimuli for this work, but additional experiments using stimuli structured specifically to probe these different within and between-category relationships could help clarify these issues.

Follow up studies might use RSA to directly compare the representational structure of LLM *embeddings* across layers (during task performance, as a query is processed) and the model’s *behavior* or task outputs. This would provide further insight into LLM knowledge representations and reasoning, and could illuminate processes related to LLM hallucinations.^55^ A suitable analogy might be adapting a psychological experiment that measures behavior to a cognitive neuroscience study that measures neural processing during task performance (via EEG or fMRI). This approach could query how representational structure changes throughout the model’s architecture and into deeper levels of processing (again similar to work by Cichy^40^ and Carlson^27^ studying representations across neural regions). This would require white box access to model activations or the ability to readout responses across layers for every query, which may be difficult for some frontier models where these are not made available. Our work takes a small step in this direction, examining Ada embeddings in the context of GPT model performance, though correspondence was quite low with actual GPT model outputs (at most, *r*_*s*_ = 0.445 with GPT-5-nano ratings). Ada embeddings are but one high-level embedding that does not allow us to probe the progression or change in any representational structure throughout the model, which future work could better address. Similarly, comparing model ratings (rather than embeddings) with human neural responses across the visual or semantic processing pathway might shed additional light on LLM computation, cognition and reasoning.

Pairwise rating tasks, RSA and related techniques can be used in the service of increasing alignment between AIs and humans. One approach involves incorporating human-like representational knowledge as an objective function to improve alignment during training or fine-tuning (and some promising initial work is being done using this approach^56^), or fine-tuning models specifically to emulate human behavior in cognitive testing.^57^ Some modifications to our tasks and prompts were able to improve alignment between LLM responses and human data, but these strategies may not scale and were posed primarily as empirical questions to better characterize Turing experimentation. More direct approaches aimed at improving alignment could be realized by incorporating objective functions that account for pairwise dissimilarity ratings provided by humans (see recent reviews^11^ for summary and discussion). A particularly useful direction could be to develop models that can be aligned not only to the representational space of the general public, but also to that of experts in a particular area. Here, distinctions among specific within-versus-between category representations for a set of items could be useful analytical distinctions. Generating the rich, stable individual differences observed in human participants, rather than the prompt- and sampling-driven variability characteristic of current LLMs, remains an open question that behavioral methods like ours are well-positioned to track as models continue to develop.

This initial work points to what could be a generalizable Human-LLM alignment task based on pairwise ratings that allowed us to systematically compare multiple language models ranging from seven to at least hundreds of billions of parameters across text and image tasks. GPT-5-mini and Claude Sonnet 4.5 achieved the highest correlation with human semantic judgments (as high as *r*_*s*_ = 0.776 for text, *r*_*s*_ = 0.696 for images). While encouraging, more work remains for fully aligning AI models with human behavior at the group and individual level, both within the context of this task, and in concert with others. Indeed, capturing the inter-individual variability of human behavior is still an outstanding issue among the LLMs evaluated here, and no model adequately captured this dimension of behavior while delivering high group-level alignment with human ratings. Methods like the one studied here help populate a toolbox of approaches, often building on cognitive science and psychology, which can help us understand these novel complex systems.

The pairwise rating method adapted here for LLM evaluation comprises a quantitative framework for helping to measure the alignment between human and artificial intelligence across input modalities. This method leverages an established cognitive science method and enabled us to describe some strengths and limitations of current LLMs: group-level semantic alignment but poor encapsulation of individual differences, slightly reduced alignment when processing images. This approach bridges cognitive science and AI evaluation, offering a potentially scalable method that can assess how artificial systems encode knowledge and align with human cognition.

### Limitations of the study

There are a number of limitations to this study that are well-suited for follow-up in future work. First, our main goal was to develop a generalizable method for querying LLM behavior as a tool to understand Human-LLM alignment, reliability, and explainability. These experiments involved a set of well-studied stimuli (words, images etc.) and relied heavily on previously collected or publicly available datasets. However, these materials, like all stimulus sets, are not exhaustive, are limited in scope and may have other shortcomings and biases.^30^^,^^51^ This of course stems in large part from their being developed with human testing in mind, and thus would be subject to practical constraints on a human participant’s time and patience. A more comprehensive set of stimuli to fully probe LLM behavior and knowledge will require additional development. This might include scaling up behavioral RSA testing stimuli to operate over more sentences, paragraphs, audio clips or movies, as well as using stimuli that can better target expertise, emotions or personality dimensions. Ultimately, the findings reported here are specific to concrete object concepts and may not generalize beyond that domain. Validation using datasets comprising more diverse stimuli (including additional objects, more abstract concepts, emotionally valenced items and specialized domains) is needed, and is well suited to follow up work.

A related limitation is that our human comparison cohort comprised a small sample of participants (*n* = 8), and future work is needed to more fully understand some of the patterns observed here, especially with respect to inter-individual variability. As discussed, pairwise rating tasks are burdensome to carry out due to the time and effort required of participants. Nonetheless, obtaining behavioral ratings for a larger number of stimuli from a larger number of human participants will be useful for grounding and expanding future explorations of LLM knowledge. Borrowing the large-scale testing structure of Hebart and colleagues^47^ could be useful in future endeavors for obtaining these data. Indeed, the work by Du and others^46^ shows this can be an effective approach. Pairwise ratings are a powerful tool in psychological research for probing knowledge and perceptual representations, but can be prohibitively time and resource intensive. For example, they require all pairs of items in the probe set to be rated (or at least all unique pairs of non-identical items), and the number of trials required in these studies increases substantially with each new item added to the set.^45^^,^^58^ While there are some more efficient paradigms that can approximate pairwise ratings,^47^^,^^58^^,^^59^ LLMs could eventually be integrated into paradigm and stimulus development to test, norm or automatically generate near-human-quality similarity ratings without the time, money or effort required to elicit ratings from human participants. This could accelerate some aspects of psychological research and allow for a more rapid search of optimal paradigms, stimuli or psychologically useful feature spaces (as suggested by Dickson and colleagues^45^).

Future work might also explore behavioral RSA approaches with respect to different kinds of behavioral context or adapt it to more naturalistic interactions. Indeed, one potential limitation of these initial experiments is that they often compared representations of words and images presented in isolation (without context). This may have disadvantaged some models that do not support a chat-prompt interface and explicitly rely on the surrounding linguistic context for word representations (e.g., the BERT family of models). However, it is clear that humans can compare semantic relations among words in isolation,^25^^,^^27^^,^^60^ so this method of comparison has some obvious validity. Nonetheless, future work could benefit from querying ratings for stimuli within a more naturalistic (e.g., interactive or conversational) context. Similarly, an important next step will be assessing pairwise rating tasks relative to more diverse kinds of behavioral tasks or outputs (potentially beyond or in addition to the pairwise ratings studied here).

## Resource availability

### Lead contact

Requests for further information and resources should be directed to and will be fulfilled by the lead contact, Mattson Ogg (mattson.ogg@jhuapl.edu).

### Materials availability

This study did not generate new unique reagents.

### Data and code availability

• All data have been deposited and are publicly available as of the date of publication at https://doi.org/10.17605/OSF.IO/FPB8Z.
• All original code has been deposited and is publicly available at https://doi.org/10.17605/OSF.IO/FPB8Z as of the date of publication.
• Any additional information required to reanalyze the data reported in this study is available from the lead contact upon request.

## Acknowledgments

We acknowledge support from the Independent Research and Development (IRAD) Fund from the Research and Exploratory Development Mission Area of the Johns Hopkins Applied Physics Laboratory. We thank L. Robert Slevc and his laboratory for collecting and sharing the human behavioral data and we thank Will Coon for helpful comments and discussions while drafting this manuscript.

## Author contributions

Conceptualization, M.O. and M.W.; methodology, M.O., R.B., J.S., C.R.R., and M.W.; investigation, M.O., J.S., and R.B.; writing – original draft, M.O.; writing – review and editing, M.O., R.B., J.S., C.R.R., and M.W.; funding acquisition, C.R.R. and M.W.; resources, M.O. and R.B.; supervision, C.R.R. and M.W.

## Declaration of interests

The authors declare no conflicts of interest.

## Declaration of generative AI and AI-assisted technologies in the writing process

During the preparation of this work, the authors used Claude (hosted by Anthropic) and Chat GPT (hosted by OpenAI) in order to update some of the verbiage and language in this report. After using this tool or service, the authors reviewed and edited the content as needed and take full responsibility for the content of the publication.

## STAR★Methods

### Key resources table

**Table:** 

REAGENT or RESOURCE	SOURCE	IDENTIFIER
**Deposited data**		

Human Behavioral Data	University of Maryland, College Park	https://doi.org/10.17605/OSF.IO/FPB8Z

**Software and algorithms**		

LLM Behavioral Testing Code	Johns Hopkins Univeristy Applied Physics Laboratory	https://doi.org/10.17605/OSF.IO/FPB8Z

**Other**		

LLM Behavioral Data	Johns Hopkins University Applied Physics Laboratory	https://doi.org/10.17605/OSF.IO/FPB8Z

### Experimental model and study participant details

Data from 8 participants who completed all trials of the semantic relatedness task in the original study by Carlson and colleagues^27^ were used in these analyses. See the original paper for full details. Information on the sex or gender of the human participants was not available and thus was not analyzed. This was not a clinical trial and human participants were not allocated to separate sub-groups. The University of Maryland Institutional Review Board approved that study and all participants provided informed consent prior to participation.

We elicited responses from different versions of OpenAI’s Generative Predictive Transformer (GPT) models^1^^,^^5^ hosted on the Azure cloud computing platform via the API (version “2023-03-15-preview” for GPT-4o models and version “2023-05-15” for all other models) and LangChain (version 0.1.0). The following GPT models were selected: GPT-3.5 Turbo Model with a 16k context window (“gpt-35-turbo-16k,” model version “0613,” referred to as “GPT-3.5”), a GPT-4 (text-only) model (“gpt-4,” model version “1106-preview,” referred to as “GPT-4”), a GPT-4-Vision model (“gpt-4,” model version “vision-preview,” referred to as “GPT-4-Vision”), and GPT-4o (“gpt-4o,” model version “2024-08-06,” referred to as “GPT-4o”) and GPT-4o-mini model (“gpt-4o-mini,” model version “2024-07-18,” referred to as “GPT-4o-mini”). For each experiment run (i.e., simulated participant) the model was initialized using a specific temperature value, and otherwise used default parameters. Unless stated (i.e., during specific follow up experiments where temperature values were swept over a set: 0.01, 0.7, 1 and 1.5) a temperature value of 1.0 was used throughout. For GPT-4-Vision models, 4096 max tokens were specified.

The GPT family of models have been among the most popular and widely used LLM tools and are thus an important class of models to understand. However, this generation of GPT models are closed source and API updates may pose challenges for reproducibility. Thus, we also evaluated a series of open-source models using the Ollama platform (also via LangChain). These quantized models were all run locally on a personal computer (2021 16-in M1 Macbook Pro with 16gb of memory), so in most cases these were run using smaller instantiations (7 to 14 billion parameters) for better throughput. This service provided access to Gemma (7b, 430ed3535049), Gemma-2 (9b, ff02c3702f32), Gemma-3 (12b, f4031aab637d), Phi-3-medium (14b, 1e67dff39209), Phi-4 (14b, ac896e5b8b34), Mistral (7b, 61e88e884507), Solar (10.7b, 059fdabbe6e6), Llama-2 (7b, 78e26419b446), Llama-2-uncensored (7b, 44040b922233), Llama-3 (8b, 71a106a91016), Llama 3.1 (8b, 46e0c10c039e), for our text experiments. These models were also re-initialized for each experiment and run using a temperature of 1.0.

The most recent generation of models was run via portkey (version 2.1.0) without LangChain. Each model was accessed via one of a few host services (Llama-4: “us.meta.llama4-maverick-17b-instruct-v1:0”, Claude Sonnet 4.5: “us.anthropic.claude-sonnet-4-5-20250929-v1:0”, Claude Haiku 4.5: “us.anthropic.claude-haiku-4-5-20251001-v1:0” all via Amazon Bedrock, Gemini-2.5-Flash-Lite via Google Cloud Platform, and GPT-5.2, GPT-5-mini, and GPT-5-nano all via Open-AI). Other large open-source models (available via Ollama, e.g., GPT-OSS 20b and 120b), large open-source mixture of experts (available via Ollama, e.g., Qwen-3), and reasoning models (Claude Opus) were considered but not run due to long run-times that would not have been feasible in the current formulation of these experiments. See Supplemental Cost and Run-Time Analysis in the Supplemental Material for additional details.

### Method details

#### Baseline neural network models

Baseline text model embeddings were obtained from public sources. From OpenAI, text embeddings were extracted from the Ada model (‘text-embedding-ada-002’ version 2, referred to as “Ada”) using the same software infrastructure as the GPT-4 models above. GloVe^61^ embeddings were obtained from an online repository (version: Common Crawl 840B tokens, 2.2M vocab, cased, 300d vectors: https://nlp.stanford.edu/projects/glove). Finally, two BERT variants from https://huggingface.co^62^ along with their tokenizers were used: a standard BERT model (“bert-base-uncased”^63^) as well as a larger variant (“albert-xxlarge-v2”^64^) that has been shown to align well with human neural responses.^65^ For each of the baseline text models, we extracted embeddings for each of the 67 words in our text stimulus set and then computed the cosine similarity between the model embeddings for each pair of words.

Two AlexNet models published along with the Ecoset dataset^41^ were used as baselines in our image rating experiments. One variant of these models was trained on the original ImageNet Large Scale Visual Recognition Challenge (ILSVRC) 2012 data (denoted “AlexNet ILSVRC2012”) while the other variant was trained on the Ecoset data (denoted “AlexNet Ecoset”). These specific model architectures achieved the best correspondence with human behavioral data in the experiments conducted by Mehrer and colleagues.^34^ Specifically, versions of the model that were trained on the Ecoset data were found to produce image classification models whose embeddings aligned better with human neural and behavioral data than models trained on ILSVRC 2012. Model variants initialized with “training seed 01” were used for each Ecoset and ILSVRC2012 model. Embeddings from layer 7 of both models’ responses to each image (rescaled to 224 by 224 resolution) were extracted and cosine distances between these embeddings from each image in the stimulus sets was used to populate the models’ DSMs. A gallery of all the DSMs generated for these experiments (averaged at the group level where appropriate) can be found in Figure S1.

#### Word similarity rating task

We compared human and LLM responses on a task judging the semantic relatedness of words based on the experiments and data from Carlson and colleagues^27^ (who obtained the appropriate ethical approvals from their institution at the time of collection, see references^27^ for further details). In this task, participants judged the semantic relatedness of a set of 67 words (see Table S1), which correspond to a subset of well-studied image stimuli depicting common objects.^32^^,^^48^ From the original data of Carlson and colleagues,^27^ we retained data from 8 participants who completed all three sessions of the task. On each trial participants were presented with a pair of words and asked to rate (using a slider) how semantically related the two objects were. The slider position was converted into a value between 0 and 50 that was recorded and analyzed (these values were re-scaled from 0 to 100 for comparability with the LLM outputs). Each participant rated each pair of words (one unique word order for each pair) for a total of 2211 ratings. Note, data from these stimulus pairs were mirrored to fill out the opposite ordered pairs for DSM visualizations, but these mirrored entries were not otherwise included in our analyses.

The task for the LLMs was modelled as closely as possible on the task administered to the human participants, with minor modifications to accommodate model responses, and to minimize errors (see Supplemental Experiment and Prompt Example 1 in the Supplemental Material). After an initial prompt introducing the task (and where applicable, the LLM participant’s surname and honorific), each trial prompted the LLM to respond with a number from 0 to 100 to characterize the relatedness of a pair of words.^66^ Following Aher and colleagues’^12^ original Turing Experiment formulation, in some experiments the LLM was assigned to assume a participant identity using a surname and honorific. This information was included at the beginning of each prompt when addressing the model. Surnames (Snyder, Smalls, Rodriguez, Olson, Nguyen, Kim, Jeanbaptiste, Garcia) and honorifics (Ms., Mr., Dr.) were drawn from a representative sample taken from the larger set used by Aher and colleagues,^12^ with the addition of the ‘Dr.’ honorific. The crossing of each surname with each honorific produced a cohort of 24 simulated participants for each of our word rating experiments. When the experiment was run for the LLM without any participant identifiers, all surnames and honorifics were removed from the prompts thus not invoking any specific identity for that participant’s run. LLM participants rated all possible pairs of items (including both orders of unique items) for a total of 4489 trials. The order of the word pairs was shuffled for each participant. An example portion of one of the LLM experiments is provided in Supplemental Experiment and Prompt Example 1 in the Supplemental Material. Note that for some combinations of the Kim, Nguyen and Jeanbaptiste surnames involving the objects pineapple, woman and cow, trials queried to GPT-4o and GPT-4o-mini were flagged by the OpenAI content filter (presumably by mistake). In such cases those trials were skipped, but future work examining differences between models with and without guardrails or content filters would be well suited to follow up work.

#### Image similarity rating task

We adapted the word similarity rating task to accommodate relatedness ratings for images corresponding to each object. We selected two sets of exemplar images for each object (i.e., for each word stimulus) from public databases. One set of stimuli comprised a set of images depicting each object in the real world that was then cropped and presented on a gray background (using the study materials posted online by,^48^ which are similar to previous stimuli,^32^ these are referred to as the “Carlson-Image” stimuli since their behavior ratings were used for analyzing neural responses to these images). This is a well-studied stimulus set in the field of cognitive neuroscience but it has numerous shortcomings as noted in previous discussions.^51^ Thus, another set of more natural object images that included representative backgrounds was drawn from the THINGS database,^49^^,^^50^ wherein a single exemplar was selected from the set of images within categories corresponding to each object label. Note, matching categories existed for 55 of the 67 objects across the Carlson and THINGS datasets. Thus, all comparisons with models or responses derived from the THINGS dataset were constrained to this subset of 55 objects where THINGS and the original Carlson and colleagues^27^ stimulus sets overlap. Note that a small number of these image stimuli (mostly THINGS stimuli depicting the ‘hair’ and ‘ear’ object classes) might occasionally fail for some models because they tripped the OpenAI content filter (ostensibly by mistake), which required us to curate our THINGS image set to arrive at a stimulus set that was usually successfully processed (Table S1 lists the image files used).

All images were resized (to a resolution of 150 by 150), converted to base64 and packaged into a dictionary for presentation to the model via the API. See Supplemental Experiment and Prompt Example 2 in the Supplemental Material for a depiction of task instructions and an example of how this experiment proceeded for a representative participant. This again resulted in 4489 ratings for the 67 Carlson Images or 3025 ratings for the 55 THINGS images. These analyses were sometimes restricted to comparisons among the 55-stimulus set for comparability but results for the full Carlson-Image dataset are reported in Table S2. The order of the image pairs was shuffled for each participant. Because image processing for some models (e.g., for GPT-4-Vision) was more expensive, image experiments were run for a reduced number of simulated participants (crossing surnames: Garcia, Nguyen, Olson, and Smalls with honorifics Ms. And Mr.) for a total of 8 simulated participants for each experiment run.

The human data for our image rating experiments comprised behavioral embeddings generated by a large number of leave-one-out ratings for images from the THINGS dataset (see the original publication^47^ for details). This embedding was obtained from a model that was able to accurately reproduce human behavioral judgements on this task (at the noise-ceiling with respect to the human behavioral data). We retained the embedding corresponding to each of the 55 THINGS object classes (that overlapped with the Carlson classes) and computed the cosine distances between them.

We found the DSMs produced by some vision models (e.g., GPT-4-Vision) to be very sparse and wondered if this was related to the vision or text processing modules. Indeed, Yuksekgonul and colleagues^67^ suggest that vision-language models can perform poorly on relational understanding and linking tasks. To compare this model’s native image processing capabilities to its text processing capabilities, another set of experiments was run where the LLM participant was first asked to provide a description of each image, and then on subsequent trials, the model rated the similarity of the images based solely on the text descriptions it had just provided. This is similar to the approach of Marjieh and colleagues,^43^ except we used a single model instantiation for the entire experiment, rather than generating descriptions with one model and comparing text embedding distances using another model. Thus, images were distilled to text descriptions and then ratings were made based only on the text descriptions. Since recent models (e.g., GPT-4o) were capable of natively processing both text and images, this allowed for cross modal analyses of that model’s specific semantic representations. A summary depiction of this task for a representative participant is provided in Supplemental Experiment and Prompt Example 3 in the Supplemental Material. Note that the “man” and “apple” stimuli were removed from the image description task of the “Carlson-Image” stimuli for the GPT-4o models since they tripped the model’s content filter.

#### Quantification and statistical analysis: representational similarity analyses

To analyze alignment between models and humans on these pairwise rating tasks, the responses among the participants for each model (or among humans) were averaged to a group-level DSM followed by a Spearman rank correlation among the flattened, group-level dissimilarity matrices. These primary statistical analyses followed recommended practices in the field,^11^^,^^38^ relying on non-parametric tests for assessing alignment.^68^^,^^69^^,^^70^ For completeness, we evaluated all pairwise Spearman correlations among model systems at the group level with a stringent Bonferroni correction applied to assess statistical significance (using a 2,415 and 5,671 comparison correction among models that assessed the 67-item Carlson dataset and the 55-item THINGS dataset, respectively, see Tables S2 and S3) against a null hypothesis of no correlation between the two DSMs (inter-model Pearson correlations can be reviewed in Tables S4 and S5). Group level comparisons were evaluated using robust non-parametric Wilcoxon tests. To evaluate inter-subject agreement, we computed correlations among individual participant’s dissimilarity matrices. Two-way intraclass correlation coefficients for agreement over the ratings of each model were also computed using the ‘irr’ package in R. Finally, individual-level human-LLM alignment was assessed via correlation between individual LLM participant DSMs and DSMs from individual human participants.

The pattern of results for the baseline models and SPoSE embeddings was consistent across the choice of distance metric, and all other model comparisons relied on behavioral distance metrics obtained via rating scales. The ranking of models returned consistent top-performing models across tested distance metrics and correlation measures (see Tables S4 and S5). Inspection of the response distributions (Figure S2) indicates that high-performing models operated over broadly similar rating scales, suggesting that differences in absolute scale did not drive the observed alignment patterns.

Throughout these experiments, we observed a small number of non-compliant trials similar to previous work,^13^^,^^60^ where the LLM provided a verbose reply (ignoring prompted instructions to reply only with a number), replied that they were an AI agent and not a participant in our study (ignoring the task prompt), or replied that a query triggered a content filter. Content filter issues mostly pertained to the GPT-4-Vision models, and we preselected image stimuli that minimized these errors as much as possible. GPT-4o had some issues with pineapple, cow and woman ratings for the Kim, Nguyen and Jeanbaptiste surnames, which were skipped. Finally, some open-source text models had difficulties with certain words (e.g., ‘gun’ or ‘woman’ for Llama-2 or Gemma). Some open-source models also provided verbose or formulaic replies (e.g., Mistral almost always explained its reasoning regardless of our prompts), which were parsed, cleaned or (where necessary) removed from further analysis.

Filtered or non-compliant trial issues arose primarily from earlier models (rates of dropped trials: Llama2-uncensored 15.45%, Gemma 8.97%, Llama-2 3.17%, Mistral 1.25%, GPT-4 1.01%), and for all other models these accounted for less than 1% of trials, with many recent models encountering no dropped trials whatsoever. Ostensibly random, non-systematic problematic trials were handled on a per-participant basis: responses for the stimulus pairs on those trials were censored only when that specific participant’s data was compared with another participant’s data. Trials were aggregated for group level analysis by aggregating the rest of the trials for that pair excepting empty trial pairs. Systematic errors, where a given model refused to process a particular stimulus (e.g., ‘gun’ or ‘woman’ for Llama-2 or Gemma), were handled by-model, removing those specific empty cells at the group level when comparing that model’s group-averaged DSM to another DSM (see the gallery of DSMs in Figure S1). Thus, we mitigated the impact of missing trials as much as possible isolating them only to analyses of those models.

## Supplemental information

Document S1. Figures S1–S4 and Methods S1–S4

Table S1. Stimuli from all datasets

Table S2. Representational alignment (measured by Spearman correlation between DSMs) across models for the set of 67 object words

Table S3. Representational alignment (measured by Spearman correlation between DSMs) across models for the set of 55 object words/images

Table S4. Representational alignment (measured by Pearson correlation between DSMs) across models for the set of 67 object words

Table S5. Representational alignment (measured by Pearson correlation between DSMs) across models for the set of 55 object words/images

## Footnotes

Supplementary data related to this article can be found online at https://doi.org/10.1016/j.isci.2026.116400.
