# Supplementary material for: A flexible behavioral method for measuring human and artificial intelligence alignment using representational similarity analysis
Source: iScience. 2026 Jun 23;29(7):116400. doi: 10.1016/j.isci.2026.116400 (PMC13320333; doi:10.1016/j.isci.2026.116400)
Supplement: Document S1. Figures S1–S4 and Methods S1–S4 [file mmc1.pdf]

**iScience, Volume *29***

## Supplemental information

# A flexible behavioral method for measuring human and artificial intelligence alignment using representational similarity analysis

**Mattson Ogg, Ritwik Bose, James Scharf, Christopher R. Ratto, and Michael Wolmetz**

# Supplemental Figure 1

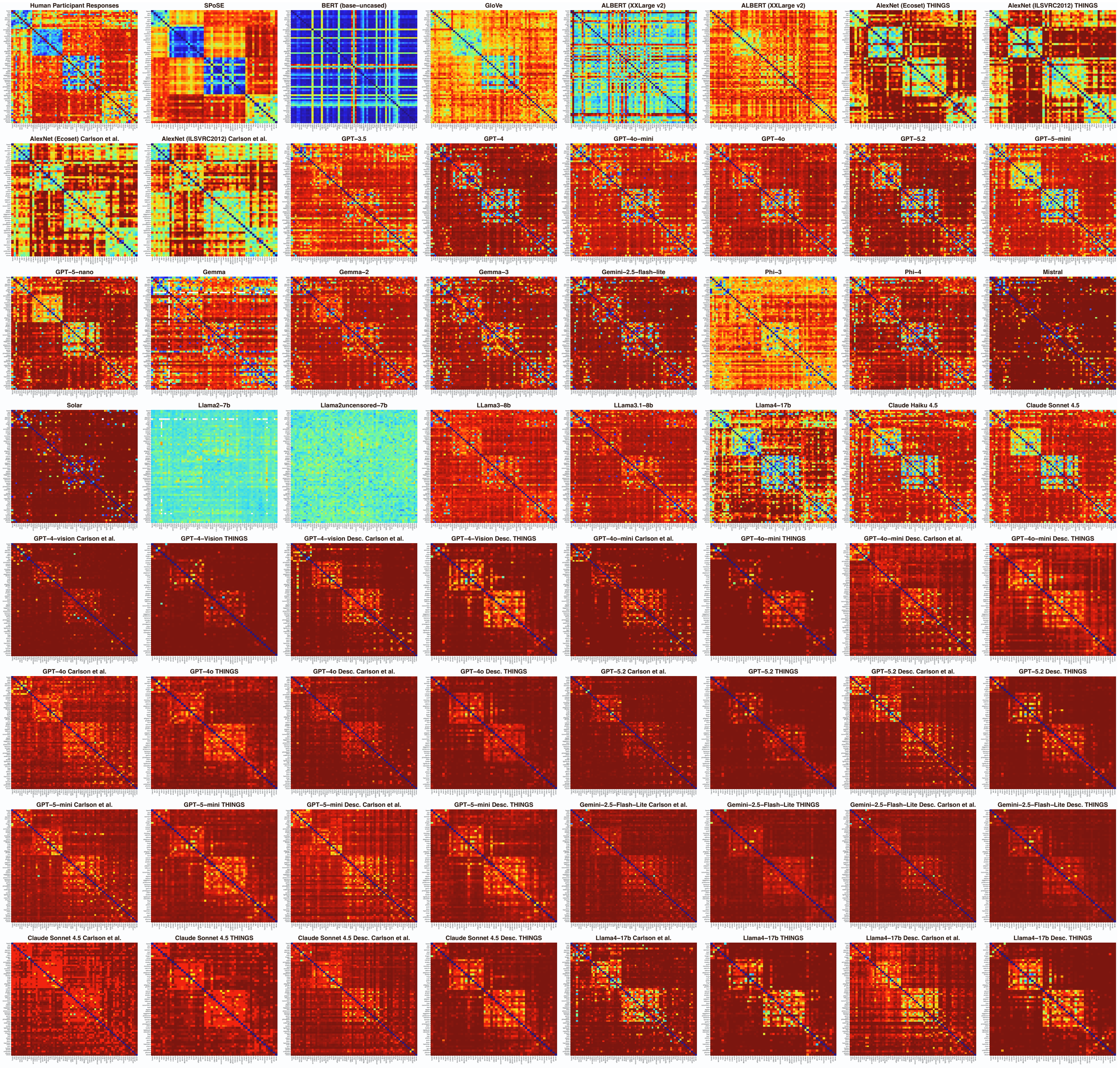


*Supplemental Figure 1* A gallery of the group-averaged DSMs generated for each model used in these experiments. Similarity ratings were converted to a distance metric and normalized to between 0 and 1 for visualization. Human ratings were only provided for one half of the dissimilarity matrix, but these responses were mirrored and the diagonal was filled for visualization.

# Supplemental Figure 2

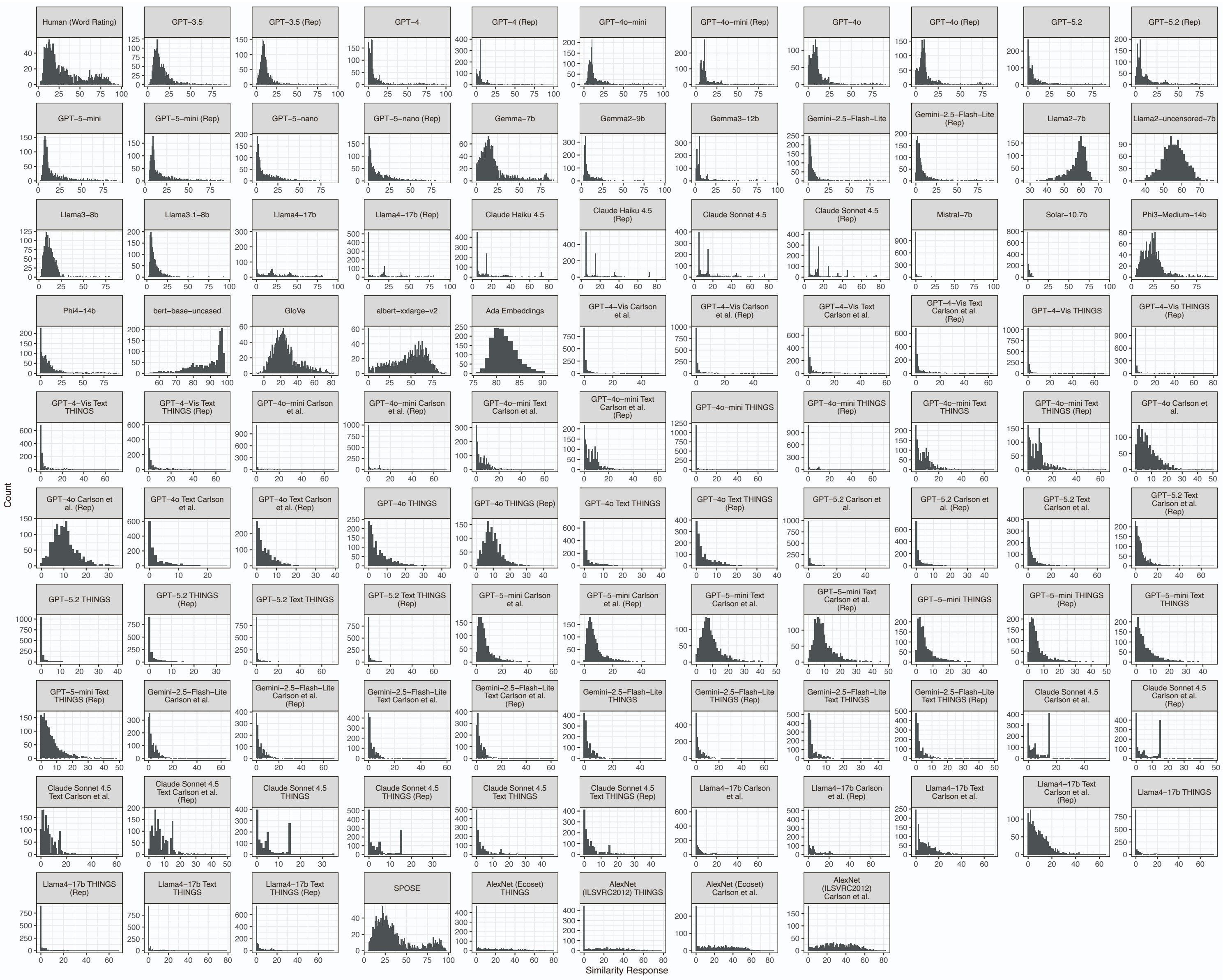


*Supplemental Figure 2* Distributions of similarity responses for all pairwise ratings or comparisons (including across participant cohorts where available) for each model.

# Supplemental Figure 3

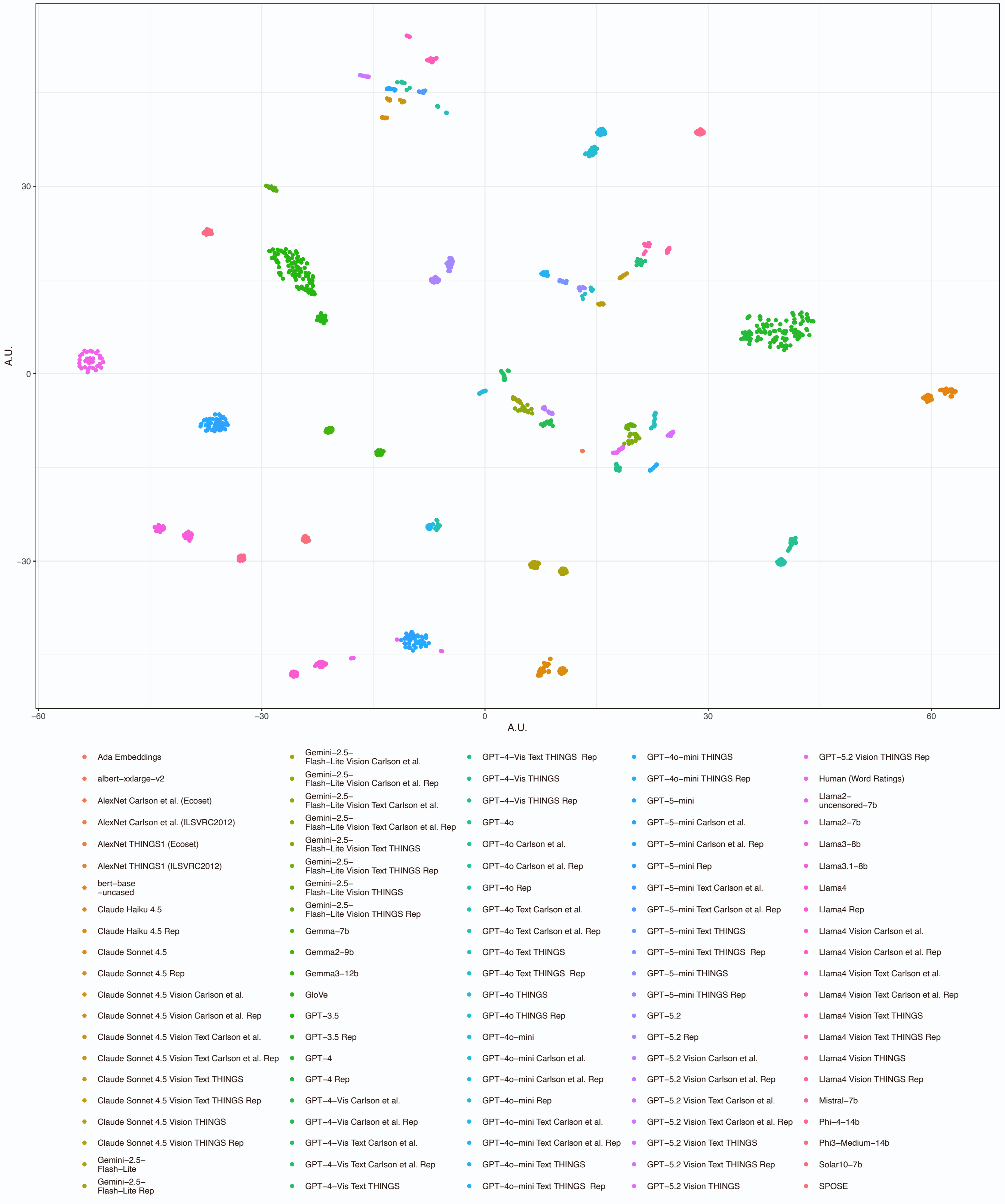

*Supplemental Figure 3* Low-dimensional visualization of the participants from each study relative to the common 55-item stimulus set. Flattened dissimilarity ratings are projected into low-dimensional space using T-SNE (from the ‘Rtsne’ package in R; using PCA, perplexity = 30, 5,000 max iterations). We computed these on one half of the reduced set of stimulus pairs for the 55 items in the image rating experiments to accommodate all models from the vision experiments. For this visualization we filled any missing values (i.e., where the LLM did not produce a response) with the midway point of the rating scale (50).

# Supplemental Figure 4

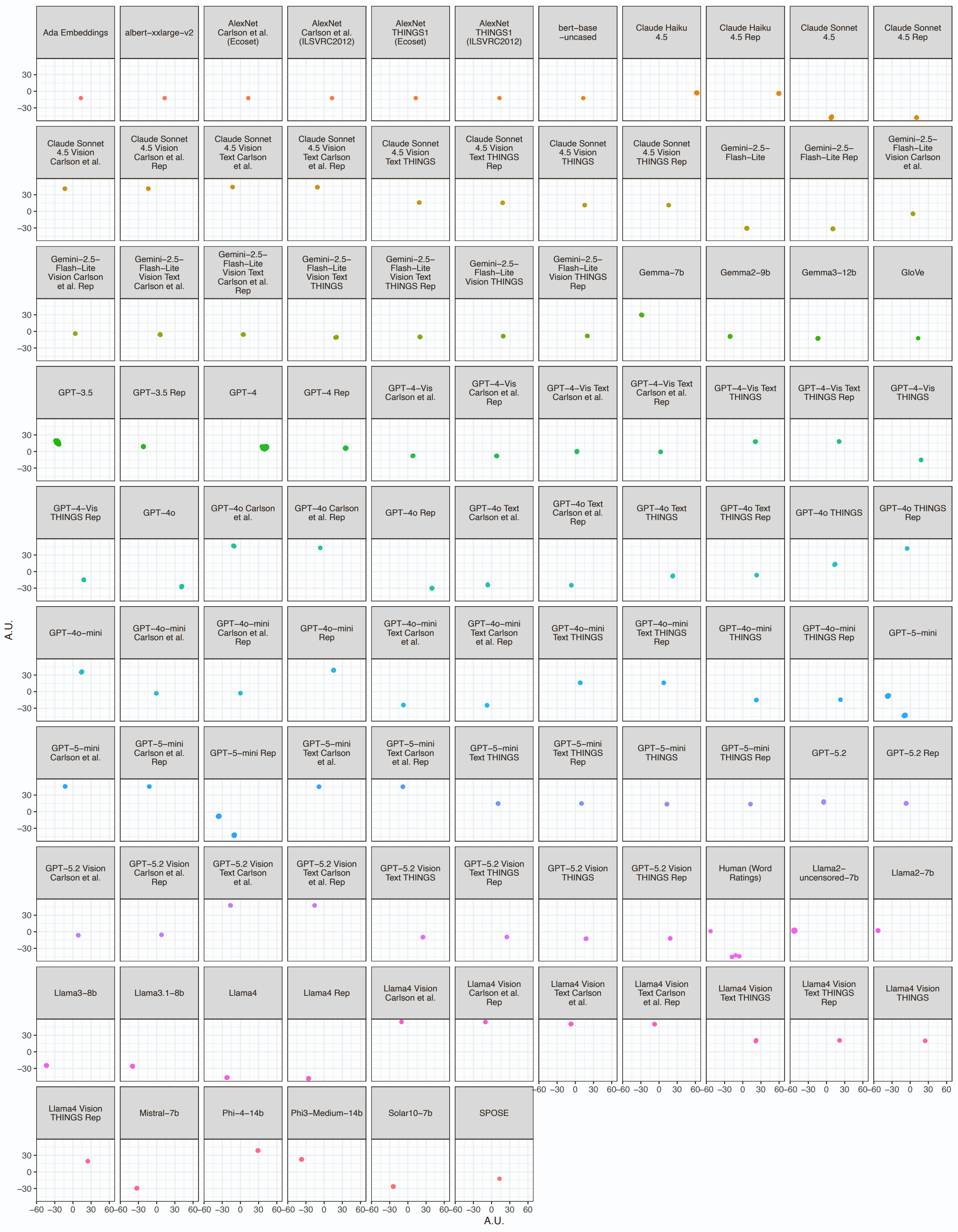

*Supplemental Figure 4* Low-dimensional visualization of the participants from each study relative to the common 55-item stimulus set. Flattened dissimilarity ratings are projected into low-dimensional space using T-SNE. This figure presents the same data as Supplemental Figure 3 but is broken out here into facets to more clearly see the locations of the data for each model.

***Supplemental Methods S1***

This is an example excerpt of an experiment run for the word similarity judgement experiment indicating the kinds of prompts we administered and the LLM's responses. Brackets were not included in the prompt, but denote portions of the prompt that were removed (and capitalization and punctuation updated as appropriate) for experiments where no participant identity was provided. General instructions and an introduction to the task were presented to LLM participants via an initial prompt similar to the initial introductory screens of the computer interface used for human participants. Instructions were presented this way for parity with the human behavioral paradigm regardless of whether a given LLM may or may not have maintained these initial instructions in memory on subsequent trial prompts.

**Experimenter (Initial Prompt):** "Hello, imagine [your name is Ms. Garcia,] you are participating in a psychology experiment. In this experiment, a number of word pairs will be displayed on the screen. Your job is to determine how RELATED each of the word pairs is on a scale from 0 to 100. 100 being very related, 0 being very unrelated. There are a number of possible ways for word pairs to be related. For the purposes of this experiment, please keep the following connections in mind when rating the word pairs:

If the two words mean the same thing, or can be used in similar ways. For example, "boat" and "ship".
If one of the words is a type of the other word. For example, "cat" is a type of animal.
If one of the words is a part of the other word. For example, "bark" is a part of a tree.
If the two words are ones you might use together in a sentence, or that might occur in similar contexts. For example, "river" and "canoe" or, "cook" and "soup."

Please do not use the spellings, or sounds, of words to make your decisions. Even though words rhyme or are spelled similarly, does not mean they are related to one another. This experiment is interested in the relation of word MEANING."

**GPT-4 Participant Response:** "As Ms. Garcia in this hypothetical psychology experiment, I understand that I am to rate the relationship between word pairs based on their semantic relationships rather than their phonetic similarities. Let's proceed with the experiment. Please provide me with the pairs of words, and I will rate their relatedness on a scale from 0 to 100 according to the guidelines given."

**Experimenter (Trial 1):** "[Ms. Garcia,] please rate how related the two words "zebra" and "house" are on a scale from 0 to 100. 100 being very similar, 0 being very dissimilar. Please respond with just a number."

**GPT-4 Participant Response:** 0

**Experimenter (Trial 2):** "[Ms. Garcia,] please rate how related the two words "path" and "path" are on a scale from 0 to 100. 100 being very similar, 0 being very dissimilar. Please respond with just a number."

**GPT-4 Participant Response:** 100

**Experimenter (Trial 3):** "[Ms. Garcia,] please rate how related the two words "garlic" and "radish" are on a scale from 0 to 100. 100 being very similar, 0 being very dissimilar. Please respond with just a number."

**GPT-4 Participant Response:** 40

## *Supplemental Methods S2*

This is an example excerpt of an experiment run for the image similarity judgement experiment using the stimuli from Carlson and colleagues (2014) indicating the kinds of prompts we administered and the LLM's responses. Brackets were not included in the prompt, but denote portions of the prompt that were removed (and capitalization and punctuation updated as appropriate) for experiments where no participant identity was provided. General instructions and an introduction to the task were presented to LLM participants via an initial prompt similar to the initial introductory screens of the computer interface used for human participants. Instructions were presented this way for parity with the human behavioral paradigm regardless of whether a given LLM may or may not have maintained these initial instructions in memory on subsequent trial prompts.

**Experimenter (Initial Prompt):** "Hello, [imagine your name is Mr. Smalls,] you are participating in a psychology experiment. In this experiment you will rate how similar different pairs of images are. Each image depicts a common object. Your job is to determine how SIMILAR each of the image pairs are on a scale from 0 to 100. 100 being very related, 0 being very unrelated. Use the entire range of the scale when making similarity ratings. The images may not be an exact match but could still be quite similar to each other! We are interested in a continuous, overall measure of similarity."

**GPT-4 Participant Response:** "Okay, I understand the task. I will carefully examine each pair of images and rate their similarity based on the criteria provided. I will use the entire range of the scale from 0 to 100 to give a precise measure of how similar I find each pair to be."

**Experimenter (Trial 1):** "[Mr. Smalls,] please rate how similar the two images are on a scale from 0 to 100. 100 being very similar, 0 being very dissimilar. Please respond with just a number. Use the entire range of the scale when making similarity ratings. The images may not be an exact match but could still be quite similar to each other! We are interested in a continuous, overall measure of similarity."

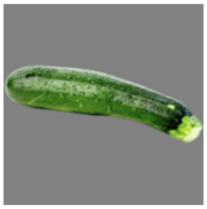 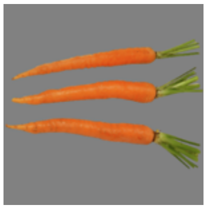

**GPT-4 Participant Response:** 10

**Experimenter (Trial 2):** "[Mr. Smalls,] please rate how similar the two images are on a scale from 0 to 100. 100 being very similar, 0 being very dissimilar. Please respond with just a number. Use the entire range of the scale when making similarity ratings. The images may not be an exact match but could still be quite similar to each other! We are interested in a continuous, overall measure of similarity."

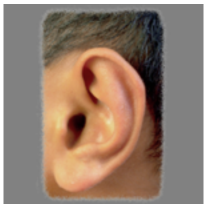 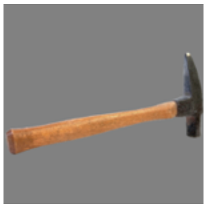

**GPT-4 Participant Response:** 0

**Experimenter (Trial 3):** "[Mr. Smalls,] please rate how similar the two images are on a scale from 0 to 100. 100 being very similar, 0 being very dissimilar. Please respond with just a number. Use the entire range of the scale when making similarity ratings. The images may not be an exact match but could still be quite similar to each other! We are interested in a continuous, overall measure of similarity."

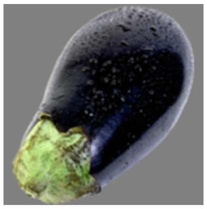 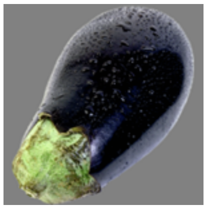

**GPT-4 Participant Response:** 100

### *Supplemental Methods S3*

This is an example excerpt of a run of the image rating experiment based on text descriptions using the stimuli from the THINGS database but using text descriptions as the inputs for pairwise ratings. Brackets were not included in the prompt, but denote portions of the prompt that were removed (and capitalization and punctuation updated as appropriate) for experiments where no participant identity was provided. General instructions and an introduction to the task were presented to LLM participants via an initial prompt similar to the initial introductory screens of the computer interface used for human participants. Instructions were presented this way for parity with the human behavioral paradigm regardless of whether a given LLM may or may not have maintained these initial instructions in memory on subsequent trial prompts.

**Experimenter (Initial Prompt):** "Hello, [imagine your name is Mr. Nguyen,] you are participating in a psychology experiment. In this experiment, you will describe a number of images and then you will rate how SIMILAR each pair of those images is based on your descriptions. First a number of images will be displayed on the screen and you will describe what you see in each. Next, you will use those descriptions to rate how SIMILAR each pair of those images is on a scale from 0 to 100. 100 being very related, 0 being very unrelated. Use the entire range of the scale when making similarity ratings. The images may not be an exact match but could still be quite similar to eachother! We are interested in a continuous, overall measure of similarity."

**GPT-4 Participant Response:** "As Mr. Nguyen, I will follow the instructions provided and carefully observe each image displayed on the screen. For each image, I will provide a detailed description of what I see, including any objects, colors, shapes, and other notable features.

Once I have described all the images, I will then move on to the similarity ratings. I will thoughtfully compare each pair of images and use my descriptions to determine how similar they are to eachother. I will take into account any shared characteristics, as well as any differences, and assign a rating on the scale from 0 to 100.

I understand that the images may not be identical, but I will focus on the overall similarities between them. I will use the entire range of the scale to make my ratings, with 100 indicating a very strong similarity and 0 indicating no similarity at all. I will approach this task with an open mind and aim to provide accurate and honest ratings based on my perceptions of the images.

**Experimenter (Description Trial 1):** "[Mr. Nguyen,] please describe this image. Do not include any historical details."

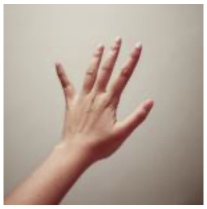

**GPT-4 Participant Response:** "The image shows one human hand with five fingers, including a thumb, slightly spread apart. The skin tone is fair, the nails are short and unpainted, and there is no visible jewelry or tattoos. The hand is positioned against a neutral, light grey background."

**Experimenter (Description Trial 2):** "[Mr. Nguyen,] please describe this image. Do not include any historical details."

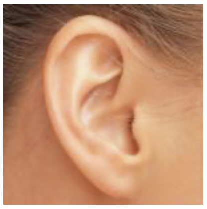

**GPT-4 Participant Response:** "The image shows a close-up of a human ear. The ear appears to be attached to a person with light skin, but the rest of the person's face or body is not visible. The ear has a typical structure with an outer curved rim called the helix, a small protrusion called the tragus, and an opening leading to the ear canal. There are no visible piercings or jewelry on the ear. The ear is

clean and appears to be healthy. The background of the image is not visible, so the focus is solely on the ear.”

**Experimenter (Description Trial 3):** “[Mr. Nguyen,] please describe this image. Do not include any historical details.”

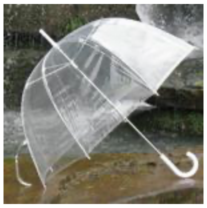

**GPT-4 Participant Response:** “This is a transparent umbrella with a white handle. It is open and resting on a wet surface, most likely due to rain. There appears to be water droplets on the umbrella, indicating that it is currently being used or has been used recently to protect against rain.”

...

**Experimenter (Rating Trial 1):** “[Mr. Nguyen,] please rate how similar the two images described below are on a scale from 0 to 100. 100 being very similar, 0 being very dissimilar. Please respond with just a number. Use the entire range of the scale when making similarity ratings. The images may not be an exact match but could still be quite similar to eachother! We are interested in a continuous, overall measure of similarity.

Description of Image 1: The image shows a close-up of a human ear. The ear appears to be attached to a person with light skin, but the rest of the person's face or body is not visible. The ear has a typical structure with an outer curved rim called the helix, a small protrusion called the tragus, and an opening leading to the ear canal. There are no visible piercings or jewelry on the ear. The ear is clean and appears to be healthy. The background of the image is not visible, so the focus is solely on the ear.

Description of Image 2: The image shows one human hand with five fingers, including a thumb, slightly spread apart. The skin tone is fair, the nails are short and unpainted, and there is no visible jewelry or tattoos. The hand is positioned against a neutral, light grey background.”

**GPT-4 Participant Response:** 20

**Experimenter (Rating Trial 2):** “[Mr. Nguyen,] please rate how similar the two images described below are on a scale from 0 to 100. 100 being very similar, 0 being very dissimilar. Please respond with just a number. Use the entire range of the scale when making similarity ratings. The images may not be an exact match but could still be quite similar to eachother! We are interested in a continuous, overall measure of similarity.

Description of Image 1: This is a transparent umbrella with a white handle. It is open and resting on a wet surface, most likely due to rain. There appears to be water droplets on the umbrella, indicating that it is currently being used or has been used recently to protect against rain.

Description of Image 2: The image shows a close-up of a human ear. The ear appears to be attached to a person with light skin, but the rest of the person's face or body is not visible. The ear has a typical structure with an outer curved rim called the helix, a small protrusion called the tragus, and an opening leading to the ear canal. There are no visible piercings or jewelry on the ear. The ear is clean and appears to be healthy. The background of the image is not visible, so the focus is solely on the ear.

**GPT-4 Participant Response:** 0

## *Supplemental Methods S4*

We wanted to evaluate the cost of the GPT series of models. Pairwise behavioral data are time consuming and thus costly for humans to generate. Given that these models (especially GPT-4) could reasonably approximate human responses, we wanted to understand how cost effective these simulated participants might be relative to human participants. We calculated the price of running trials for a full dissimilarity matrix (4489 rating trials) for each GPT participant using Langchain's callbacks. We caveat that these estimates can vary depending on the specific prompt, the system's output, proximity to the target endpoint and network traffic on a given day.

A full experiment run for a GPT-3.5 participant cost approximately $0.86 and took approximately 20 minutes per participant, while a run for a GPT-4 participant was more expensive at $8.75 and took about an hour and 10 minutes per participant. A GPT-4o participant cost $0.75 and took approximately 30 minutes to run through the experiment while a GPT-4o-mini participant cost $0.05 and took approximately 20 minutes to run through the experiment. We expect this compares favorably with human data collection, which Carlson and colleagues (2014) report required three separate testing sessions for one unique set of item pairs (2211 trials). Conservatively estimating $10 per hour for their time, and 3 hours total for a human to rate half the full pairwise set of 67 items, this is an acute increase in efficiency.

Overall, the vision models were slower and often more expensive to run than text-only models, especially the GPT-4 versions. Current instantiations of visual reasoning models may not be as scalable as text-based models. However, runtime was considerably more efficient for GPT-4o, and we expect efficiency to continue to improve as the technology improves. A full experiment run of a GPT-4-Vision participant to obtain a complete set of pairwise ratings for the limited set of 55 image stimuli (3025 trials total) cost approximately $55 and took 13 hours to complete. Note however, this required uploading both images to a server for each trial, which is a larger transmission than text-alone. Rating images converted to text descriptions was also cheaper (approximately $20 per experimental run of a GPT-4-Vision participant) and faster (approximately 8.5 hours per experimental run) than directly comparing each image. We note, however these improvements likely stem from the lower bandwidth requirements for transmitting text data compared to image data. The GPT-4o models were much cheaper and faster costing $3.86 and $7.76 for GPT-4o and GPT-4o-mini, respectively (note GPT-4o-mini is priced at a higher rate for processing images than GPT-4o), and participant run took an hour to an hour and ten minutes to complete.
